# Experiences of a new work model among primary care staff when treating patients with hypertension – a qualitative study

**DOI:** 10.1080/02813432.2025.2507282

**Published:** 2025-05-23

**Authors:** Rebecka Quester, Per Hjerpe, Malin Östman, Susanne Andersson

**Affiliations:** ^a^ Region Västra Götaland, Närhälsan Bollebygd Healthcare Centre, Bollebygd, Sweden; ^b^ General Practice/Family Medicine, School of Public Health and Community Medicine, Institute of Medicine, Sahlgrenska Academy, University of Gothenburg, Gothenburg, Sweden; ^c^ Regionhälsan R&D Centre, Skaraborg Primary Care, Skövde, Sweden; ^d^ Regionhälsan R&D Centre, Fyrbodal Primary Care, Vänersborg, Sweden

**Keywords:** Hypertension, primary healthcare, qualitative research, nurse’s role, task shifting

## Abstract

**Objective:**

Hypertension care requires considerable resources from primary healthcare, and efficient work models are desirable both to improve treatment outcome and to ease staff workload. This study’s objective was to describe how healthcare staff experienced the implementation of a new nurse-led work model for hypertension care.

**Methods:**

Qualitative content analysis was used for data analysis. Digitally conducted interviews with 14 physicians, nurses and managers from six of the eleven primary healthcare centres participating in an intervention to improve hypertension care in the Västra Götaland region. The intervention included training of healthcare professionals in a new nurse-led team work model using standardized protocols for treatment and follow-up.

**Results:** The intervention was appreciated, even requested, by both nurses, physicians and managers. The clearly defined division of tasks in the team provided structure, safety, and eased the workload. Treatment was streamlined and the work was perceived as more professional and stimulating. However, implementation of the new work model, including task shift, required close cooperation between nurses and physicians, trust and dedication. Implementation failed if the staff turnover was high, or if management support lacked. Personal qualities, judgement, experience and learning by cooperating with each other, were highlighted as important additional factors for competence and professionally performed hypertension care.

**Conclusion:** Healthcare can benefit from this intervention, but manager support and involvement of both nurses and physicians are crucial factors for successful implementation. Structured protocols cannot replace experience and personal qualities but provide appreciated support and increased safety.

## KEY POINTS

Introducing a work model including task shifting between physician and nurse might be a way to streamline hypertension care.

- Support from the management was crucial for a successful implementation of the new nurse-led work model.
- Clearly defined teamwork eased the workload and streamlined treatment, but required involvement of both nurses and physicians.
- In addition to structure and protocols, personal qualities and experience were considered important for a professionally performed hypertension care.

## Introduction

International guidelines recommend that a majority of people with hypertension receive a diagnose and treatment [1,2]. This is a major task for our healthcare systems, as approximately every third adult worldwide is affected [3]. Hypertension care in Sweden is traditionally performed in primary care, led by a physician, with follow-up performed by a nurse. However, these routines vary greatly between different primary healthcare centres (PHCCs) [4]. In the last decades, multiple studies have implied that hypertension care led by nurses is associated with more content patients, higher achievement of treatment target [5–7] and less cases of stroke [8]. Nurses performing tasks traditionally handled by physicians, task shifting, has also been highlighted as a way to solve staffing shortages and to streamline healthcare [6,9]. The latest ESC hypertension guidelines recommend task shifting as necessary to meet the great need for hypertension care [2].

A recent article of ours [10] describes an intervention in 2018, involving a structured nurse-led hypertension care model. The work model was implemented at eleven volunteering PHCCs in different parts of the region Västra Götaland. The intention with the intervention was to achieve a higher percentage of patients with hypertension reaching treatment targets, by streamlining and standardizing the care process with written routines and a clearly defined teamwork [11]. We could show signs of changed treatment and improved treatment results for the participating PHCCs compared to non-participants. It is, however, of great importance to know how a changed work model is experienced by the working staff, how the team work around the patient is perceived and if obstacles are experienced during implementation [12]. It is also important to take the implementation success into account when evaluating treatment results from an intervention [13]. Studying the staff’s experiences is therefore necessary for understanding what challenges healthcare staff meet, what aspects that may be important for succeeding with implementation, and what results to expect when introducing new work models in hypertension care [12,13].

This study aims to describe how the staff at the participating PHCCs experienced the implementation and practice of the new work model, which involved teamwork and an altered division of tasks and responsibilities between the physician and a nurse placed in charge.

## Methods

### Design

A qualitative design with tradition based on an effort to develop an understanding of the human, lived experience was used in our study, as it was appropriate when studying experiences [14]. The interviews were analysed unprejudiced, without a theoretical framework, to identify patterns and meaning in the data [14,15].

### Participants and context

The participants were nurses, physicians and PHCC managers from six of the eleven PHCCs participating in the voluntary hypertension improvement intervention in 2018 [10]. The intervention involved education at five whole-day seminars, spread over a year and a half. Standardized protocols were provided to support a nurse-led work model, in which a nurse in charge for the hypertension team was responsible for both diagnosis, risk stratification, drug titration and check-ups, with supervision from a physician [10]. The work model could be adapted for both patients with single diagnosis and multimorbidity. How the new work model was used varied between participants. During implementation, the participants were able to discuss their progress and get support at the seminars [12]. Two years after the start of the intervention, all participating PHCCs were invited to this follow-up study. The invitation was sent by e-mail to the PHCC managers, who forwarded it. Participants from six PHCCs chose to participate (see Table 1). The included PHCCs were of varying sizes, located in both cities and rural areas, differed in grade of hypertension treatment target fulfilment and represented both private and public care.

**Table 1.** Profession, gender and PHCC number of participants in the six interviews, separately.

Interview (labelled A-F)	Profession	Gender (f/m)	PHCC (numbered 1-6)
A	Nurse	m	3
	Nurse	f	2
	Nurse	m	5
B	Nurse	f	6
	Nurse	f	6
C	Nurse	f	4
	Nurse	f	1
D	Physician	m	5
E	Physician	f	2
	Physician	f	2
F	PHCC manager/nurse	f	2
	PHCC manager/nurse	f	3
	PHCC manager/physician	f	1
	PHCC manager/physician	f	1

### Data collection

The participants were interviewed between November 2021 and April 2022, in groups, pairs or individually, depending on their ability to participate. The interviews were 30 to 60 min long and performed digitally, with all participants and interviewers able to see and hear each other. The recorded interviews were transcribed verbatim and covered in total 100 pages (Times New Roman, size 12, line spacing 1.0) when transcribed. All participants gave written informed consent.

The interviews were led by one of the authors (SA), with long experience of moderating interviews. One or two of the other authors participated at each interview for notetaking and complementary questions. An interview guide with open-ended questions was used (see Supplementary material). Examples of questions were: How come your PHCC joined the intervention? If there have been any changes in how you work due to the intervention, please tell us about them? Can you elaborate on that a bit?

### The authors’ backgrounds

The first author (RQ) is a PhD student and general practitioner (GP) with experience in routine hypertension care in primary care. Author PH, PhD and GP, has experience in conducting quantitative hypertension research and working with hypertensive patients in primary care. PH was involved in designing and implementing the staff training intervention in this project in collaboration with, among others, the author MÖ, an experienced district nurse and PhD with experience in conducting qualitative research on patients with cardiovascular disease. Author SA, PhD and diabetes nurse, has worked many years especially with patients with hypertension and diabetes, and has experience conducting qualitative research in patients with long term diseases in primary care.

### Data analysis

Data was analysed with qualitative content analysis. The method is used to identify patterns, variations, similarities and differences. The manifest analysis describes visible, concrete parts of the text and the latent analysis describes and interprets on a deeper level with higher abstraction [14,15].

In the first step of the data analysis, all authors separately read through all interviews several times, to achieve an overall understanding of the content. The text was divided into meaning units by the authors separately and then discussed together. From there, authors RQ and SA continued the analysis with condensing and labelling the meaning units with codes that were concrete and close to the text. To maintain the core meaning, the authors repeatedly returned to the original text. Codes that did not answer the research question were excluded. Excluded text parts consisted of for example initial presentations and occasional parts where the interview clearly had diverted from the subject, such as an in-depth discussion concerning calibration of measure devices.

RQ formed subcategories by grouping codes similar to each other, then discussed and adjusted the subcategories together with SA (see Table 2). Finally, the subcategories were sorted and abstracted into categories by RQ and SA together, supported by MÖ. At this point, a theme with a higher level of abstraction and interpretation was described and formulated.

**Table 2.** Example from the analysis process.

Meaning unit	Condensed meaning unit	Code	Subcategory	Category
Because you’ve got clear instructions and you know what to do, treatment and so on, it has made treating and working with hypertension patients easier.	The structure clarifies tasks, which facilitates working with hypertension	Structure facilitates hypertension work	Clearly defined workflows facilitate work and create security	Implementation of the work model can benefit both staff and healthcare outcome
And to feel secure in the fact that we know what we are doing too, that we don’t miss anything. We have full control.	Feeling secure that the work will be done correctly	Work quality is secured		
This also leads to, I believe, we don’t let them go until they have a blood pressure that is at a good level.	Patients are followed until the treatment goal is reached	Actively working to achieve goals	Successful, targeted treatment is standardized and allows individual adaptation	
But then, if there is someone who needs to come in and be checked a little more, if it is a little more urgent, we will try to solve it.	Patients with urgent need of extra controls will get priority	Patients with greater needs are prioritized		

Subcategories, categories and theme were revalued by RQ and SA separately and in discussions, and in relation to the original text. The text parts that had been excluded were read thoroughly, to ensure that no relevant data had been lost. Final adjustments were made and debated in the whole research group until all authors were in agreement.

### Ensuring trustworthiness

Trustworthiness in qualitative research can be assessed based on its credibility, dependability, confirmability and transferability [16]. To ensure the results’ credibility, care was taken to establish a safe atmosphere during the interviews. For example, participants were thoroughly introduced to each other and reminded of the study’s purpose and voluntariness before continuing to interview questions, and during the interview all participants were encouraged to speak up. The collected data was perceived as rich. The analysis was systematic, performed both separately and together and revalued repeatedly to also ensure the results’ dependability.

In order to secure the results’ confirmability, the research group was composed of authors with different clinical professions and experience, and throughout the analysis there was an awareness of and an openness to misunderstandings resulting from the author’s own preunderstanding. To establish transferability the process for sampling, interviewing and analysing data was described in detail (above), and data was presented in a concrete way with quotes from the participants.

## Results

During the analysis, nine subcategories were identified, forming three categories: *The intervention can meet needs – if it is prioritized in the organization*, *Implementation of the work model can benefit both staff and healthcare outcome*, and *Professional roles in close team collaboration can only develop in unison,* and one theme: *Skilful professional collaboration requires experience, practical wisdom and judgement* (see Table 3).

**Table 3.** Subcategories, categories and theme that emerged from the analysis.

Subcategory	Category	Theme
An intervention that supports learning through cooperation	The intervention can meet needs – if it is prioritized in the organization	Skilful professional collaboration requires experience, practical wisdom and judgement
Providing requested structure through credible written protocols		
Introducing a new work model requires drive, support, and resources		
Clearly defined workflows facilitate work and create security	Implementation of the work model can benefit both staff and healthcare outcome	
Successful, targeted treatment is standardized and allows individual adaptation		
Updated methods for measuring and follow-up has support in guidelines		
The nurse’s increased autonomy comes with greater responsibilities	Professional roles in close team collaboration can only develop in unison	
Task shifting between nurse and physician alters the professional relationship		
The teamwork is dependent on collaboration, confidence, and trust		

### The intervention can meet needs – if it is prioritized in the organization

There is a need for knowledge and structure in hypertension management, which the intervention can meet. However, if not given sufficient priority by staff, management and organisation, following the protocols will be difficult and implementation fails.

#### An intervention that supports learning through cooperation

The intervention’s initial training contributed to interprofessional learning, inspiration and motivation: ‘*It’s been so much fun. I feel like I have learned so much … both medical things and how to talk to patients.*’/B. The participants described the project as stimulating, affirming, exciting and meaningful. Both physicians and nurses experienced that, by collaborating through the new work model and discussing with colleagues, their knowledge about hypertension increased. They expressed a greater understanding of the importance of having appropriate knowledge and skills when working with hypertension, and an increased awareness of knowledge gaps: *‘unfortunately, I think many are poorly updated’/D*. Learning by sharing experiences and knowledge with others outside their own PHCC was especially emphasized.

#### Providing requested structure through credible written protocols

The participants experienced a need for structure when managing the large group of patients with hypertension. Structured and concise protocols based on existing guidelines were desirable for optimizing the workflow. A manager saw the need and described: ‘*we had long wait times for the patients for scheduled visits and we needed to review our workflows…*’/F. As writing own protocols at each PHCC would require too much time to be a realistic alternative, the intervention’s protocols were appreciated. The fact that they were compiled by people with long experience of working with hypertension treatment, increased the protocols’ credibility, but a risk for decreased reliability was perceived, unless they were updated regularly.

The participants described that the protocols brought structure to the work and unified all professions’ objectives for the care. Everyone could work in the same direction and get the whole picture. The protocols could also function as an educational support towards the patients. Clear protocols were particularly helpful for junior physicians and trainees, for guidance in assessment and treatment. Even experienced staff had support from the protocols in their daily work. *‘Even if you’ve worked for many years … and know exactly what you’re doing, having it [the protocol] is still such a comfort and reassurance’*/E. However, unless the protocols were known by all concerned, a risk for deviations from the structure was perceived. Having temporary staff became more difficult, though the protocol constituted a valuable support when introducing new employees. The protocols’ usability could be increased if they were compiled into quick-to-view short texts, giving treating staff a quicker overview. This was especially helpful for unexperienced staff.

#### Introducing a new work model requires drive, support, and resources

The intervention encouraged local adaptations of the protocols’ routines. The participants considered it important to be able to organize the work based on each PHCC’s conditions whilst maintaining the structure’s objective. Developing local routines required time, but sometimes high workloads and lack of time hindered development. However, an openness to adaptation was considered necessary. For example, during the Corona pandemic annual return visits were postponed or adapted to minimize the risk of infection.

The management’s encouragement and participation was considered a decisive support for successful introduction of the new work model: *‘We had one of our managers in the project as well which made it easier to reach out to the doctors’*/C. Without the manager’s consent to prioritize the new work model, carrying out the work according to the protocols was difficult. The new work model did not require more resources, but needed a redistribution of existing resources. The participants experienced that their work in the hypertension team sometimes received lower priority than other diagnosis-oriented care. One view was that it would facilitate if there was a requirement from a higher level to have a specialized nurse in hypertension, as there is in diabetes care.

Another decisive factor was someone in the workplace driving the work forward. It could be either a nurse or a doctor. A committed, motivated person who saw the structure’s benefits, or as a participant said: ‘*it takes enthusiasts and interested people to run it*’/E. Of most importance was an interest in the hypertension field. Knowledge and training could provide inspiration and generate a driving force. This was considered especially important initially during the implementation.

### Implementation of the work model can benefit both staff and healthcare outcome

If a successful implementation of the new work model is achieved, the staff experience that they can improve the quality of care by using their resources in a safer, more efficient way.

#### Clearly defined workflows facilitate work and create security

When the new work model was followed according to the protocols, a simplified handling of patients and a clarification of responsibilities in the work group was described. The clear plan promoted cooperation and action, leading to smoother work flow with fewer delays, shorter processing time and a feeling of more efficient resource use: ‘*…we’re using our resources in a better way! So more people benefit from them*’/F. The defined treatment steps with ready-made drug choices, dosages and follow-up blood samples allowed automated handling, which saved energy. The structure also required fewer blood pressure checks, and involved a plan for every blood pressure measured.

In addition, a feeling of security was experienced when everyone knew what to do, how and by whom: ‘*It creates such a sense of security, I don’t have to second guess or double check*’/D. Since the physician had the overall medical responsibility, the protocols’ clear directions for what the nurse could decide independently was a security for both physician and nurse. A written delegation was seen as the correct and safe way to handle the responsibility handed over to the nurse, however, this was sometimes missing.

#### Successful, targeted treatment is standardized and allows individual adaptation

The participants experienced that previous hypertension treatment and handling of patients could vary depending on the physician in charge. The new work model enabled them to offer more equal care, with uniform assessment and standardized procedure regarding choice of drugs and blood pressure measuring technique. Awareness increased regarding the importance of correct measuring, as deviant values could lead to incorrect diagnosis or treatment, and also affect the PHCC’s treatment target statistics negatively. One observed consequence was that abnormal values or blood pressures measured non-standardized less often were entered in the medical record. Instead, they were remeasured to register more correct values and favour the statistics.

The new work model was considered to promote targeted treatment. ‘*You follow them [the patients] more. And push them, and say it’s new now – last time you were here, the blood pressure should have been this, but now they [the guidelines] have changed and now it should be this.’/B.* The staff’s interest in treatment targets and the PHCC’s results increased, and improved results provided inspiration and confirmation. An increased accuracy of diagnosing, earlier start of medication and lowered risk of losing patient’s follow-up was perceived. However, the possibility to make individual adaptations was regarded as important, for example, prioritizing patients with greater needs for a more rapid follow-up. Patients’ already existing medications could successfully be supplemented based on the protocols. However, in some cases successful medications had been exchanged to equivalent preparations simply to stick to the protocol’s proposed drugs. Such strict adherence to the protocol was regarded undesirable and unnecessary.

One experience was that the new work model focused more on cholesterol values than previous routines did. Consequently, more patients with hypertension received lipid treatment. This was less prioritized previously, despite support in the guidelines. ‘*We have become much more - tougher when it comes to following guidelines on blood fats and blood pressure’/B.* A dialogue arose in the team about a common strategy to meet the patient concerning cholesterol treatment. It was found easier than expected to offer cholesterol treatment. There had been initial concerns that patients would report more side effects with intensified antihypertensive and cholesterol treatment, but that was not seen.

#### Updated methods for measuring and follow-up has support in guidelines

The transition to the new work model introduced new methods for measuring blood pressure and communicating with patients. The use of ambulatory blood pressure monitoring and home monitoring increased, which modern guidelines support. Attitudes towards home monitoring became more encouraging: *‘Before, a doctor might have said: No, you shouldn’t get a blood pressure monitor at home. It will just be stressful for you … Now the doctor is like: Please, feel free, just go on and check it’/C.* Home monitoring was perceived to give patients direct feedback on their treatment and reduce the risk of overtreatment, but it involved new educational challenges in instructing patients. The participants noticed that some patients seemed uncertain and more anxious about home monitoring.

Various forms of digital communication for follow-up also increased. Digital communication was sometimes practical, but for certain patients or situations, traditional forms of follow-up were preferred.

### Professional roles in close team collaboration can only develop in unison

New responsibilities in the team alters the professional roles and require close collaboration, a transformation dependant on mutual trust and agreement between nurse and physician in order to develop.

#### The nurse’s increased autonomy comes with greater responsibilities

The new work model involved increased responsibilities for the nurse in charge. However, being entrusted to independently make decisions and titrate drug treatment based on the protocol also made the nurse’s work more interesting and satisfying: ‘*We can right there and then inform them about which medicine we’ll use, how it works and why we follow up with samples and so on - and it feels more professional, too, than saying I’ll call you*…’/A. In addition, the new work model brought more and new administrative tasks, such as assessing blood sample results. This increased administration could feel burdensome, and using the time for meeting patients had sometimes been preferred. Balancing hypertension work with other tasks at the clinic, and maintain competence without limiting the work, could be a challenge. Some preferred variety, while others preferred to work only with hypertension, if possible, and specialize further.

The new work model gave the nurse in charge an important, central role, and skill enhancement. A clearer professional role brought increased security for the nurse, who also experienced more trust from both patients and colleagues: ‘*other nurse colleagues, they ask me if they have questions … instead of going directly to the doctor*’/A. Attaching responsibility to a specific person also gave personal characteristics increased importance for the work’s performance. Thus, an experienced nurse was considered an invaluable asset, but concentrating the competence to one person was more vulnerable to absences. Working standardized still required a use of common sense and professionally performed individual evaluations, as everyone could not be treated equally.

#### Task shifting between nurse and physician alters the professional relationship

Increased continuity and a feeling of increased patient safety was described as a result of the task shift, as patients met the same nurse every time. Following up of dose changes prescribed by the physician further contributed to the nurse’s overall control of the treatment. An increased focus on lifestyle habits was experienced, as the nurse had time to talk about preventive measures based on the patient’s needs. A physician’s visit, in contrast, was often occupied by competing health problems, leaving an insufficient part of the visit to actual blood pressure control, evaluation of treatment and lifestyle habits: ‘*they [the patients] would gather things, when they would come for their annual check-up to the doctor … blood pressure often became almost like an afterthought in the visit… Because there might be knees that needed attention, too*’/A. The staff experienced that the patients seemed satisfied with the nurse having time for them and planning the treatment together, which increased their involvement, awareness and motivation.

The new work model gave the nurse tasks that were previously performed by the physician’s, with the physician in a more consulting role. Delegating treatment and monitoring of long-term illnesses to a nurse was perceived as a good model, if there was no other reason for a physician’s assessment. One experience, however, was that older patients with hypertension could benefit from being followed by physicians, as they tended to have more complex health problems. Some of the participants described a routine to transfer patients above a certain age from the nurse to the physician. It was preferred if the nurse primarily took on new patients not yet used to be treated by the physician.

As a consequence of the new work model, coworkers outside the team met fewer patients for hypertension control only, and a possible risk of declining experience was perceived. Nurse colleagues had occasionally expressed uncertainty, even a lost trust to carry out hypertension related tasks. Physicians in training getting less experience in handling patients with hypertension was another perceived risk. There had been concerns that the physicians’ work could become heavier when uncomplicated patient cases were delegated to nurses: ‘*The discussions have certainly come up, should we let go of all the easy cases and just focus on the heavy ones? … But at the same time, we don’t have enough time as it is now*’/E. When work progressed according to the protocols, however, the physicians described a noticeable and appreciated relief. Time was freed to focus on multi-morbid patients and complex patient cases. Even nurse colleagues expressed feeling relieved as patients could be referred to the nurse in charge of hypertension.

#### The teamwork is dependent on collaboration, confidence and trust

Working in teams was perceived to gather competence around more complicated cases, and the new work model required well-functioning collaboration in the team. Previous experience of teamwork facilitated cooperation. Allowing physicians and nurses to have a dialogue regularly was essential, and an established time for consultation was considered valuable. The nurse could plan treatment and suggest preparations and dosages in prescription documents, but a physician needed to issue the prescription. If the physician was unfamiliar with the new work model or for other reasons omitted the protocol, the nurse could initiate a dialogue and reflection with support of the protocol. ‘W*hen you present it like, “these are the latest recommendations”, they [the physicians] can actually change their assessment and prescription based on what we say*.’/B. This created conditions for consensus on treatment.

Occasionally the nurse, especially if new to the work model, experienced uncertainty concerning how to apply routines in individual patient cases: ‘*when you’re unsure about what to do and whether the cases fall inside or outside the score … I find it really helpful to be able to discuss it with a doctor.*’/A. Medical assessments too difficult to handle by the nurse were necessary to be able to hand over to a physician. In addition to a patient-responsible physician for each patient, it was considered important to have a physician with overall responsibility for hypertension treatment at the PHCC, including the introduction and maintenance of the new work model.

One perceived risk was that the new work model would discontinue if the physicians lacked time set aside for the responsibilities and administrative tasks that came with it. Prescriptions, assessments and answering questions could be experienced as a burden if there was no time, and handing over responsibility to the nurse without time for collaboration was not experienced safe. Workplaces with high physician turnover experienced difficulties in assigning a physician responsible for hypertension treatment, and to fully implement the new work model. For example, some patients continued treatment according to previous routines, while others were handled according to the protocols. With such partial implementation, care was perceived as unequal.

Although the protocols had been presented to the physicians, they were not always followed. Establishing the protocols in the physician group was considered essential. Without a committed, present physician prioritizing nurse support, the nurse’s situation was problematic: ‘*It didn’t work there. I didn’t have a doctor backing me up*’/B. Most physicians were described as willing to follow the protocol, but their compliance and attitude varied. Occasionally, new physicians questioned the new work model’s structure, others expressed disbelief in the correctness of the protocols’ recommendations. Some older physicians were hesitant about change, and a general inertia in changing working methods was described for all staff categories. However, the nurses involved in the new work model were generally positive about following it.

### Skilful professional collaboration requires experience, practical wisdom and judgement

Through the results a recurring theme was that professional collaboration – between nurse and physician, as well as between caregiver and patient – was facilitated by a well-implemented, structured work model, but also required something more: A good judgment and a developed ability to transform theory to useful practice. To strictly follow written protocols and to have a structured routine was considered very helpful in order to increase efficiency and quality of care. However, to achieve a true first-class care, the participants highlighted the importance of personal qualities, work experience and common sense. Developing such personal qualities requires experience, which cannot be replaced by protocols or guidelines.

## Discussion

### Principal findings

The nurse-led hypertension care intervention was appreciated – even requested – by both nurses and physicians. When successfully implemented, the participants experienced improvements in teamwork, treatment effectiveness, work load and a more professional, stimulating way to work. However, implementation of the new work model, and the task shift involved, required close cooperation and understanding between nurses and physicians, and failed if the staff turnover was too high, if there were lack of resources or dedication or – importantly – the management’s support.

### Strengths and limitations

The choice of participants determines what results that can be obtained [14]. All PHCCs participating in the intervention were invited to join this study. That means, everybody with the experience we aimed to study were able to participate, which is a strength. Though participants from only six of the intervention’s eleven PHCCs chose to participate, our sample represented PHCCs with a satisfactory variation in size, location and treatment target fulfilment. The results also showed an obvious variation in degree of implementation, which implied that the sample was sufficient to generate rich data with a lot of variation.

Being a qualitative study, our results are prone to be limited by a subjectivity caused by the authors participation in creating and interpretating the data [16]. This matter has been taken into account by the authors throughout the analysis (see *Method, Ensuring trustworthiness*). The involvement of multiple researchers initially familiarizing themselves with the data independently was one way to triangulate and strengthen the results.

The knowledge that this study has generated is valuable for understanding the impact of the intervention, and it is a strength that there is an earlier quantitative evaluation of the intervention [10], as the two studies confirm and supplement each other’s results. Though the setting for this study is specific for our project’s particular intervention, some of the results should be transferable to other, similar contexts, for example other interventions or improvement works in primary care involving task shifting and/or hypertension care.

### Findings in relation to other studies

The participants described a variation in degree of implementation of the new work model at their workplaces. This is in line with the results from our earlier quantitative study, which showed a significant change in the PHCCs’ treatment patterns in line with the protocol’s recommendation, but with large variations between the different PHCCs’ results [10]. A varying degree of implementation of an intervention is common and even expected. Partial implementation can still have an important impact as the development can continue for years [12,17].

Support from the management was considered very important for succeeding with the implementation, and collaboration between nurse and physician was considered crucial for the new work model to function. An umbrella review from 2021 [6], covering both quantitative and qualitative studies on task shifting, identified supportive organisation system and collaboration among all parties as two important key elements for successful implementation. Other identified key elements were training and competence. Our results showed that both adequate knowledge and work experience were considered important factors for success.

The umbrella review [6] also concluded that task shifting from physician to nurse is associated with improved blood pressure control. The participants in our study experienced improved care and better treatment results with the new work model, though for older patients, some participants considered it better to deviate from the work model’s routine. Instead, a physician-led care was preferred, due to older patients’ often multiple health problems. To routinely transfer patients above a certain age to a physician is an example of a local adaptation of the work model at some PCCs.

The new work model’s advantages for treatment results were partly attributed to the structured written protocols. Following the protocols was assumed to result in adequate care. However, to achieve a superior care, solely following the protocols was apparently not enough. In addition, the participants experienced a need of personal qualities, work experience and common sense. These results can be compared with results from a recent study in the same Swedish region Västra Götaland but in another context, where a structured foot examination form was introduced in diabetes care [18]. The healthcare staff in that study also expressed a need of judgement, experience and knowledge, in order to verify the form.

Our results showed that increased knowledge and skill enhancement was experienced to be an important gain from the intervention and the new work model. Task shifting being experienced to be educative and skill enhancing has been described in multiple earlier studies, according to a Cochrane qualitative evidence synthesis [19]. However, the participants in our study emphasized learning not from the task shift in itself but learning by sharing knowledge and experiences with others, inside as well as outside their own PHCC, and especially the learning resulting from the teamwork that the new work model entailed. In addition, secured means for nurse and physician to communicate was described as essential for the teamwork, and trust in the team. Another newly published study from the Swedish region Västra Götaland exploring collaborative learning between entrepreneurs, healthcare professionals and patients [20] also points out trust as vital for a learning environment, and the importance of structure, a clear plan and time set aside for collaboration.

The nurse’s new responsibilities, gained skills and knowledge was on the one hand experienced as an advantage, as it led to more stimulating and professional work performance and increased competence in the team. Similar experiences have been lifted by other studies on task shifting [19]. But on the other hand, the participants in our study also saw a vulnerability in pooled competence in a single person, as it made it difficult to replace this person. A nurse with specialized skills not defined or recognized in other care contexts was also seen as problematic, compared to for example the well-established concept of a diabetes nurse.

### Clinical implications

This study has provided valuable knowledge in combination with the earlier quantitative study that evaluated the intervention’s impact on cardiovascular control [10]. Our recent study adds information about how the intervention was experienced. In addition to a positive learning, the staff experienced the new work model as effective, facilitating, stimulating and safe. In conclusion, healthcare can benefit from this intervention and the work model it promotes, as it improved both treatment results and the staff’s work experience.

Several of the participants did however not experience a complete implementation of the work model, which lead us to another important conclusion: that optimizing hypertension care is complex and requires a combination of many factors. To provide education and well written, reliable protocols is only one part. In addition, stable conditions in the workplace and the manager’s support are crucial factors for allowing functioning teamwork to develop, as are the personal qualities that our participants inquire for, in order to achieve a fully skilled professional collaboration: Good judgement and ability to transform theory to practice – which both require experience to develop. We emphasize that the individual coworker’s experience and personal qualities decisively affect the outcome of the teamwork and that experienced staff cannot be replaced without further ado. Protocols cannot replace experience – but bring valuable support, regardless the staff’s experience.

## Conclusions

Healthcare can clearly benefit from this intervention and the work model it promotes, as both treatment results [10] and the staff’s work experience improved. However, manager support and involvement of both nurses and physicians are crucial factors for successful implementation. The outcome also depends on the staffs’ personal qualities and experience, which structured protocols cannot replace – but they can increase safety and provide well appreciated support regardless the staffs experience.

## Supplementary Material

Interview_Guide_Rebecka_Quester_Experiences_of_a_new_work_model_among_primary_care_staff.docx
