# Supplementary material for: Experiences of a new work model among primary care staff when treating patients with hypertension – a qualitative study
Source: Scand J Prim Health Care. 2025 May 23;43(4):795–804. doi: 10.1080/02813432.2025.2507282 (PMC12632213; doi:10.1080/02813432.2025.2507282)
Supplement: Interview_Guide_Rebecka_Quester_Experiences_of_a_new_work_model_among_primary_care_staff.docx [file IPRI_A_2507282_SM0421.docx]

**Interview Guide**

*Primary care staff’s experience of a new way of working regarding the treatment of high blood pressure in primary care.*

**BACKGROUND INFORMATION**

- Profession?
- How many years of experience in the profession/in primary care/with hypertension care?
- Specific education in hypertension care?

**QUESTION AREAS**

**Please, tell us about how your PHCC came to join the project?**

- How did you get information about the project?
- What made you interested in participating?

**Can you describe how you worked with patients with hypertension before the project?**

- Describe your routines, for example a newly discovered patient with hypertension?
- How could it happen, give examples
- How were the tasks distributed?
- Who did what? Structure?
- How was information reported between you?

**If there have been any changes in how you work due to the intervention, please tell us.**

- Tell us if something has changed?
- In that case, describe what has changed?
- Is that a positive thing? Less positive? Negative?

**About the future, tell us how you will you continue your work from now?**

- Thoughts about the future?
- How do you reason as a manager/nurse/physician?

**Do you have any thoughts or suggestions for anyone else who could be relevant?**

**Is there anything else you would like to add to this interview?**

**Follow-up clarifying questions:**

What were you thinking when you told me that ….

What is your experience concerning ….?

Can you elaborate on that a bit?

Can you give an example on that?
